# The prevalence of chronic obstructive pulmonary disease in patients with spondyloarthritis compared to the general population in the southernmost region of Sweden: a case–control study

**DOI:** 10.1007/s10238-024-01335-x

**Published:** 2024-04-10

**Authors:** Petros Zamout, Sofia Exarchou, Ankita Sharma, Carl Turesson

**Affiliations:** 1 Section of Rheumatology, Örnsköldsvik Hospital, Örnsköldsvik, Sweden; 2 Rheumatology, Department of Clinical Sciences, Malmö, Lund University, Jan Waldenströms Gata 1B, 205 02 Malmö, Sweden; 3 Department of Rheumatology, Skåne University Hospital, Malmö, Sweden

**Keywords:** Spondyloarthritis, Chronic obstructive pulmonary disease, Comorbidities

## Abstract

Spondyloarthritis (SpA) has been associated with comorbidities, e.g., cardiovascular disease. However, little is known about the relation between SpA and chronic obstructive pulmonary disease (COPD). The aim of the study was to compare the prevalence of COPD in SpA to the general population. Patients with prevalent SpA in Skåne, Sweden, on December 31, 2018, were identified based on diagnostic codes in a regional register on primary care, secondary outpatient care and inpatient care. Population-based controls (5 per SpA case) were matched for age, sex and municipality. The base case definition for COPD required at least two prior visits with a registered COPD diagnosis. Stricter definitions included dispensed prescriptions for COPD and a COPD diagnosis made by a specialist in lung medicine. The prevalence of COPD in patients with SpA and controls, overall and stratified by sex and age, and the corresponding prevalence ratios, were estimated. A total of 3571 patients with SpA (51% male, mean age 53 years) were compared to 17,855 matched controls. The prevalence of COPD in patients with SpA was 37.8/1000, with a prevalence ratio compared to controls of 1.03 (95% CI 0.85–1.24). There were no significant differences in COPD prevalence between patients with SpA and controls in men or women, in any of the age groups, or in analyses using stricter definitions of COPD. In this regional study including data from primary care, the prevalence of COPD was not increased in patients with SpA compared to the background population.

**Supplementary Information:**

The online version contains supplementary material available at 10.1007/s10238-024-01335-x.

## Background

Spondyloarthritis (SpA) describes a group of chronic inflammatory diseases that share genetic, pathophysiologic and clinical features [1]. SpA is nowadays seen as a spectrum of phenotypes that includes patients with predominantly axial disease (axial SpA-axSpA) with sacroiliitis and spinal syndesmophyte formation, and those with predominantly peripheral disease (peripheral SpA-pSpA) with enthesitis synovitis and dactylitis [2]. AxSpA is further classified as radiographic axSpA (r-axSpA, formerly ankylosing spondylitis, AS) and non-radiographic axSpA (nr-axSpA) depending on the presence of typical sacroiliitis on X-rays. Among patients classified as pSpA, most have been diagnosed with psoriatic arthritis (PsA), arthritis associated with inflammatory bowel disease (IBD), reactive arthritis or undifferentiated SpA. Irrespective of the phenotype, SpA frequently occurs together with other related inflammatory extra-musculoskeletal manifestations, such as anterior uveitis, psoriasis, and inflammatory bowel disease (IBD) [3–6].

In addition, SpA has been associated with morbidity from other diseases. An increased mortality has been demonstrated in AS compared to the general population [7], and patients with AS and other types of SpA have been shown to be at increased risk of cardiovascular events [8]. By contrast, there is limited information on the relation between SpA and chronic obstructive pulmonary disease (COPD). Register-based studies from Israel [9] and Taiwan [10] have suggested an increased prevalence of COPD in patients with AS compared to the general population, and a study of patients hospitalized with AS in Sweden reported an increased incidence of COPD [11]. However, to our knowledge, there have been no studies on the prevalence or incidence of COPD in patients with SpA in general.

COPD is a complex respiratory disease, characterized by irreversible airflow limitation. It is the result of airway inflammation and remodeling, and often associated with parenchymal destruction and the development of emphysema. COPD is the third leading cause of morbidity and mortality worldwide [12]. An increased risk of COPD has been found in patients with rheumatoid arthritis (RA) [13], probably partly due to smoking as a common risk factor, but also likely related to effects of chronic inflammation [14]. Insights on the prevalence of COPD in patients with SpA may help us to understand the nature of systemic inflammation in SpA, and its relation to comorbidities. Furthermore, information on which comorbidities are overrepresented among patients with SpA would be helpful for healthcare providers who see such patients in their clinical practice.

The aim of this study was to investigate the prevalence of COPD in a population-based cohort of patients with SpA, and compare it to the general population.

## Methods

### Study design

This was an observational study conducted in Skåne, the southernmost region of Sweden, (population aged ≥ 18 years 1,069,459, 528 983 men and 540,476 women, on December 31, 2018), which used prospectively collected data from population-based databases that were linked together based on the personal identity number (= personnummer), a ten digit number widely used in Sweden to identify individuals, and used by authorities, health care, schools universities, banks, insurance companies, etc. A cohort of patients diagnosed with SpA was compared to controls without SpA. Prevalent COPD was defined based on previously registered diagnoses and drug prescription. We investigated the prevalence of COPD in 2018 in patients with prevalent SpA at this time and controls. The underlying assumption is that both SpA and COPD are chronic diseases which can be considered prevalent from the first diagnosis until death.

The study was approved by the Swedish Ethical Review Authority (September 23, 2000; registration number 2020–04041, with amendment on February 16, 2021; registration number 2021–00717). As all delivered register data were pseudonymized, the investigators did not have access to personal identity information. The Ethical Review Authority decided that individual informed consent was not necessary for this study.

### Data sources

ICD-10 diagnostic codes from inpatient care, primary outpatient care and secondary outpatient care were retrieved from the Skåne Healthcare Register (SHR). The SHR is an administrative healthcare register that contains information from electronic medical records and administrative databases on healthcare consultations in the Skåne region from 1998 and later. Each single healthcare consultation to all types of healthcare professionals generates data entries that are automatically transferred to the SHR. During the period 2012–2016, 97% of the general population in the area consulted some type of health care at least once [15]. Private and public organized care is registered in the same way in the SHR, except for the diagnostic codes in private care which are not transferred to the SHR. For inpatient care, the proportion of consultations that have an assigned diagnosis has been close to 100% over the whole period. For secondary outpatient care, the proportion of consultations with a registered diagnosis has been high and increasing since 2000. The proportion of consultations with an assigned diagnosis has been lowest within primary care, although it increased markedly in 2004 when the prerequisite for consultation reimbursement was linked to the requirement for an assigned diagnosis, reaching ≥ 80% for physician visits after 2004, and close to 100% in 2017 [15].

The Swedish Prescribed Drug Register (PDR) contains data on all prescribed drugs dispensed by Swedish pharmacies since July 1^st^, 2005, with national coverage close to 100%. The register does not include data on drugs used in hospitals, and only partial data on drugs that are used in ambulatory care but are administered in day-care hospitals (e.g., intravenously administered anti-rheumatic drugs) [16]. Data from the PDR were used to identify treatment with anti-rheumatic drugs, as well as COPD medication other than glucocorticoids. Regarding COPD, we consider it unlikely that a patient would be treated for COPD at a hospital consultation, and never dispense a prescription for self-administered COPD treatment that would be registered in the PDR.

The Swedish biologics register was initiated in 1999 by the Swedish Society for Rheumatology and represents a subset of the Swedish Rheumatology Quality Register (SRQ) [17]. Studies of biologic disease modifying anti-rheumatic drugs (bDMARDs) in the SRQ are coordinated by the Anti-Rheumatic Therapy in Sweden (ARTIS) group. The SRQ includes not only prescribed subcutaneous bDMARDs, but also intravenous therapies, which are usually not captured by the PDR. According to recent estimations, the SRQ captures approximately 86% of administered bDMARDs for SpA [18]. SRQ was used as a complement to the PDR to identify intravenous bDMARD treatment.

The Statistics Sweden (SCB) is a government agency responsible for the maintenance of a number of registers (e.g., the Total Population Register–TPR or census register, since 1969) [19]. SCB data was used for information on country of birth, places of residence, level of formal education and income for SpA patients and controls.

### Patients with SpA

Patients with SpA were identified from the SHR, using predefined ICD-10 codes listed in supplementary Table 1. In order to limit the heterogeneity of the study population and focus on effects of SpA rather than those of extra-musculoskeletal manifestations such as psoriasis and IBD, we concentrated on patients that had been assigned diagnoses codes corresponding to AS (M459) and undifferentiated SpA (M460, M461, M468 and M469). We believe that this should best reflect the entities r-axSpA, nr-axSpA and undifferentiated peripheral SpA. The SpA case definition required having at least one registered SpA diagnosis from a specialist in rheumatology or internal medicine, alternatively at least 2 registered SpA diagnoses from any physician in primary or secondary care, between January 1st, 1998 and December 31st, 2018. Furthermore, they should be at least 16 years of age at first registered SpA diagnosis and at least 18 years old, alive and residing in Skåne on December 31st, 2018.

In order to assess potential misclassification, we investigated the proportions of patients with SpA and controls that had been diagnosed with RA at any time during the study period, defined as the presence of ≥ 1 consultation with a registered diagnosis with the ICD-10 codes M05 or M06 in the first diagnosis position.

### Controls

Controls from the general population were identified using SHR data, i.e., among those with at least one contact with the healthcare system at any time during the study period, in accordance with previous case–control studies based on the SHR [20, 21]. Five individuals were matched to each patient with SpA (case). Matching was performed for age at the time of first SpA diagnosis for the case (within 1 year), municipality of residence at this time, and sex. These individuals were not given a SpA diagnosis before December 31st, 2018, and were at least 18 years old, alive and residing in Skåne on December 31st, 2018.

### Identification of patients with prevalent COPD

Among the prevalent SpA cases and controls, patients that received a COPD diagnosis registered in the SHR before December 31st, 2018 were identified. In order to account for diagnostic challenges in clinical practice, we used 2 definitions of COPD, base case definition and strict case definition. Patients matching our base case definition of COPD were individuals with 2 or more registered COPD diagnoses in the SHR between 2000 and 2018, being of 18 or more years of age, alive and residing in Skåne on December 31st, 2018. Patients matching our strict case definition of COPD were individuals with 2 or more registered COPD diagnoses in the SHR between 2000 and 2018 and 2 or more dispensed prescriptions for COPD medication other than glucocorticoids between 2016 and 2018 registered in the PDR.

The ICD-10 and ATC codes used for the identification of COPD are listed in Supplementary Table 1.

In order to assess whether patterns were similar for a more valid COPD diagnosis, made by a lung specialist, we applied a sensitivity analysis. In this, only patients fulfilling the strict definition that had ≥ 1 COPD diagnosis registered by a specialist in lung medicine were included as COPD cases.

### Further information on the study population

Individuals with a history of SpA-related extra-musculoskeletal manifestations (anterior uveitis, psoriasis, inflammatory bowel disease), and selected general comorbidities (cardiovascular disease (CVD), diabetes mellitus (DM), venous thromboembolism (VTE) and arrhythmia) were identified among SpA cases and controls using ICD-10 codes and ATC codes for prescribed drugs (Supplementary Table 2). CVD and DM were included as they are common comorbidities in the general population, whereas VTE and arrythmia were selected based on previously described associations with SpA [8, 22]. Information on treatment with systemic glucocorticosteroids during the study period (described as dispensation of ≥ 1 of more prescription of a drug with the ATC code H02AB) was retrieved from the PDR. Data on treatment with DMARDs in patients with SpA were retrieved from the PDR and the SRQ register.

### Statistical analysis

Descriptive statistics for demographics (age, sex, country of birth) and comorbidities were obtained for SpA cases and controls. Descriptives were also listed by prevalent COPD in SpA cases for these variables, SpA related manifestations and SpA related medication.

The prevalences per 1000 of COPD in patients with SpA and controls were estimated by dividing the number of COPD cases with the total number of individuals in each group, overall, by sex and by age group (< 30, 30–39, 40–49, 50–59, 60–69, 70–79, > 80 years). Estimates were obtained using the base case definition and the strict definition for COPD.

Prevalence ratios for COPD in patients with SpA vs controls were calculated for each definition of COPD, overall and by sex and age group. 95% confidence intervals (CI) for prevalence estimates and prevalence ratios were determined using the Poisson distribution ratio.

Prevalence estimates with 95% CIs for other comorbidities in patients with SpA and controls were obtained using the same methods as for COPD.

In the sensitivity analysis, the corresponding analyses of prevalences and prevalence ratios were performed using the strict definition of COPD with further requirement of ≥ 1 COPD diagnosis registered by a specialist in lung medicine.

Statistical analyses were performed using SPSS (version 25).

## Results

### Patients with SpA and controls

A total of 3571 patients with prevalent SpA were identified. With a background population of 1 069 459, this corresponds to a prevalence of SpA using this definition of 0.33% on December 31, 2018. The control group consisted of 17 855 individuals matched to the prevalent SpA cases at the identification for age, sex and municipality. The median number of consultations with a registered diagnosis in the SHR during the study period was 59 (interquartile range (IQR) 33–94) in patients with prevalent SpA, and 34 (IQR 19–60) in controls. A total of 463 patients with SpA (13.0%) and 266 controls (1.5%) had received a diagnosis of RA at any time during the study period.

Characteristics of the study population are presented in Table 1. There were no major differences in country of origin between SpA cases and controls. Diabetes mellitus was slightly less prevalent in patients with SpA (82.3 per 1000; 95% CI 73.2–92.3 vs. 98.3 per 1000; 95% CI 93.8–103.1 in controls), whereas they were more likely to have a history of arrythmia (96.6 per 1000; 95% CI 86.7–107.4 vs. 82.7 per 1000; 95% CI 78.6–87.1) or venous thromboembolism (68.3 per 1000; 60.0–77.5 vs. 55.5 per 1000; 95% CI 52.1–59.1) (Table 1).

**Table 1:** Patients with spondyloarthritis and controls. Demographics and comorbidities

	SpA	Controls
N	3571	17 855
Male sex; n (%)	1833 (51.3)	9165 (51.3)
Age; mean (SD)	53.2 (15.3)	53.7 (15.3)
Country of birth; n (%)		
Sweden	3076 (86.1)	15,409 (86.3)
Scandinavia except Sweden	92 (2.6)	285 (1.6)
Europe except Scandinavia	128 (3.6)	558 (3.1)
Other	275 (7.7)	1603 (9.0)
*Comorbidities; n (%)*		
Cardiovascular disease	31 (0.9)	128 (0.7)
Diabetes mellitus	294 (8.2)	1756 (9.8)
Venous thromboembolism	244 (6.8)	991 (5.6)
Arrhythmia	345 (9.7)	1477 (8.3)
*Treatment (ever); n (%)*		
Systemic glucocorticosteroids	1839 (51.5)	2712 (15.2)

*SpA* Spondyloarthritis, *SD* Standard deviation

### Characteristics of patients with SpA, by the presence of COPD

Among the patient with SpA, 135 fulfilled the base case definition for COPD. Compared to those without COPD, these were older and more likely to be female (Table 2). They also had higher prevalences of each of the investigated general comorbidities. Among SpA-related extra-musculoskeletal manifestations, they were more likely to suffer from psoriasis and inflammatory bowel disease, but they were to a lesser extent treated with DMARDs, in particular TNF inhibitors (Table 2).

**Table 2:** Patients with spondyloarthritis by prevalence of chronic obstructive pulmonary disease (base case definition). Demographics, comorbidities, SpA manifestations and treatment

	No COPD	COPD
N	3436	135
Age; mean (SD)	52.6 (15.3)	69.4 (11.1)
Male sex; n (%)	1778 (51.7)	55 (40.3)
*Country of birth; n (%)*		
Sweden	2959 (86.1)	118 (87.4)
Scandinavia except Sweden	88 (2.6)	4 (3.0)
Europe except Scandinavia	123 (3.6)	5 (3.7)
Other	266 (7.7)	8 (5.9)
*SpA related extra-musculoskeletal manifestations; n (%)*		
Anterior uveitis	108 (3.1)	4 (3.0)
Psoriasis	455 (13.2)	37 (27.4)
Inflammatory bowel disease	281 (8.2)	18 (13.3)
*General comorbidities; n (%)*		
Cardiovascular disease	26 (0.8)	5 (3.7)
Diabetes mellitus	255 (7.4)	39 (28.9)
Venous thromboembolism	215 (6.3)	29 (21.5)
Arrhythmia	304 (8.8)	41 (30.4)
*Treatment (ever); n (%)*		
Methotrexate	732 (21.3)	26 (19.3)
Sulfasalazine	495 (14.4)	16 (11.9)
Other csDMARD	73 (2.0)	4 (3.0)
TNF inhibitor	843 (24.5)	24 (17.8)
JAK inhibitor	29 (0.8)	1 (0.7)
IL-17 inhibitor	95 (2.8)	3 (2.2)
PDE4i	9 (0.3)	1 (0.7)
Any csDMARD	3436 (28.6)	35 (25.9)
Any b/tsDMARD	862 (25.1)	25 (18.5)
Any DMARD treatment	1249 (36.4)	40 (29.6)
Glucocorticosteroids	1741 (50.7)	98 (72.6)

*SpA* Spondyloarthritis, *COPD* Chronic obstructive pulmonary disease, *TNF* Tumor necrosis factor, *JAK* Janus kinase, *PDE4i* Phosphodiesterase-4 inhibitor, *DMARD* Disease modifying anti-rheumatic drug, *csDMARD* Conventional synthetic DMARD, *bDMARD* Biologic DMARD, *tsDMARD* Targeted synthetic DMARD

### Prevalence of COPD in patients with SpA and controls

The estimated prevalence of COPD, using the base case definition, in patients with SpA was 37.8 per 1000 (95% CI 31.7–44.7). Prevalence estimates were similar for patients with SpA and controls overall (prevalence ratio 1.03; 95% CI 0.85–1.24) as well as in separate analyses of men and women (Table 3).

**Table 3:** Chronic obstructive pulmonary disease in patients with spondyloarthritis and controls–base case definition

	SpA cases with COPD n/N	Controls with COPD n/N	SpA	Controls	COPD Prevalence Ratio (95% CI)
			COPD prevalence/1000 (95% CI)	COPD prevalence/1000 (95% CI)	
All	135/3571	657/17,855	37.8 (31.7–44.7)	36.8 (34.0–39.7)	1.03 (0.85–1.24)
Women	80/1738	353/8690	46.0 (36.5–57.3)	40.6 (36.5–45.1)	1.13 (0.88–1.45)
Men	55/1833	304/9165	30.0 (22.6–39.1)	33.2 (29.5–37.1)	0.90 (0.67–1.21)
*All–by age group (years)*					
< 30	0/219	0/990	n/a	n/a	n/a
30–39	2/479	8/2315	4.2 (0.5–15.1)	3.5 (1.5–6.8)	1.21 (0.12–7.27)
40–49	2/775	26/3905	2.6 (0.3–9.3)	6.7 (4.3–9.8)	0.39 (0.04–1.59)
50–59	21/851	99/4230	24.7 (15.3–37.7)	23.4 (19.0–28.5)	1.05 (0.63–1.69)
60–69	41/678	207 /3385	60.5 (43.4–82.0)	61.2 (53.1–70.1)	0.99 (0.69–1.38)
70–79	46/422	224/2215	104.1 (76.2–138.8)	101.1 (88.3–115.3)	1.03 (0.73–1.41)
≥ 80	23/147	93/815	156.5 (99.2–234.8)	114.1 (92.1–139.8)	1.37 (0.83–2.17)

*SpA* Spondyloarthritis, *COPD* Chronic obstructive pulmonary disease, *CI* Confidence interval; *n/a* Not applicable

In patients with SpA as well as in controls, the prevalence of COPD was higher in women than in men, and increased with higher age (Table 3). There were no significant differences in the prevalence of COPD in patients with SpA compared to controls in any age group overall (Table 3) or in analyses of men and women separately (Supplementary Table 3). The highest prevalence ratio (1.37) was observed in the group aged ≥ 80 years; although the difference did not reach statistical significance (95% CI 0.83–2.17).

As expected, the prevalence of COPD among patients with SpA when using the strict definition was lower than the base case estimate (26.3 per 1000; 95% CI 21.3–32.2) but again similar to controls (prevalence ratio 1.03; 95% CI 0.82–1.29). Patterns in analyses stratified by sex and age (Table 4) and by age separately in men and women (Supplementary Table 4) were similar to those using the base case definition for COPD, with no significant difference between SpA cases and controls in any of the subanalyses.

**Table 4:** Chronic obstructive pulmonary disease in patients with spondyloarthritis and controls–strict case definition

	SpA cases with COPD n/N	Controls with COPD n/N	SpA	Controls	COPD Prevalence Ratio (95% CI)
			COPD prevalence/1000 (95% CI)	COPD prevalence/1000 (95% CI)	
All	94/3571	455/17,855	26.3 (21.3–32.2)	25.5 (23.2–27.9)	1.03 (0.82–1.29)
Women	56/1738	247/8690	32.2 (24.3–41.8)	28.4 (25.0–32.2)	1.13 (0.83–1.52)
Men	38/1833	208/9165	20.7 (14.7–28.5)	22.7 (19.7–26.0)	0.91 (0.63–1.29)
*All–by age group (years)*					
< 30	0/219	0/990	n/a	n/a	n/a
30–39	1/479	5/2315	2.1 (0.1–11.6)	2.2 (0.7–5.0)	0.97 (0.02–17.06)
40–49	0/775	16/3905	n/a	4.1 (2.3–6.7)	n/a
50–59	16/851	60/4230	18.8 (10.7–30.5)	14.2 (10.8–18.3)	1.33 (0.71–2.32)
60–69	27/678	147/5385	39.8 (26.2–57.9)	43.4 (36.7–51.0)	0.92 (0.58–1.38)
70–79	33/422	158/2215	78.2 (53.8–109.8)	71.3 (60.6–83.4)	1.10 (0.73–1.60)
≥ 80	17/147	69/815	115.6 (67.4–185.2)	84.7 (65.9–107.1)	1.37 (0.75–2.34)

*SpA* Spondyloarthritis, *COPD* Chronic obstructive pulmonary disease, *CI* Confidence interval; *n/a* Not applicable

The sensitivity analysis, using the strict definition of COPD with further requirement of ≥ 1 COPD diagnosis registered by a specialist in lung medicine, yielded even lower prevalence estimates in patients with SpA and controls, but prevalence ratios were similar to the main analyses (Supplementary Table 5).

## Discussion

In this study, based on a regional register of primary and specialized care, the prevalence of physician-diagnosed COPD in patients with SpA was 3.8%. This estimate was not different from that in age- and sex-matched population controls. Results were similar in analyses stratified by sex, and when using stricter definitions of COPD.

COPD was more prevalent in women than in men, in the SpA subset as well as in controls. This may reflect a predominance of women among smokers in younger age groups in Sweden in later years [23] and/or women being more likely to seek care for obstructive symptoms and be diagnosed with COPD.

Patterns for the comparison between SpA and controls was largely similar across age groups. The trend toward an association between SpA and COPD among those aged ≥ 80 years should be interpreted with caution, as it did not reach statistical significance and was not observed in any other subset.

The results of the present study differ from those presented previously in studies focusing on AS. Sharif et al. [9] reported a prevalence of COPD of 4.8% in patients with AS in Israel, compared to 2.1% in their reference population. This difference appeared to be largely driven by a higher proportion of smokers in the AS population (46% compared to 18% among controls). However, they also observed a weak but statistically significant independent association between AS and COPD (odds ratio 1.22, adjusted for age, sex and smoking). In a study on the relation between AS and asthma, based on a national register in Taiwan, Shen et al. [10] reported on COPD and other comorbidities at baseline. They found a higher prevalence of COPD in patients with AS compared to controls (7.5% vs. 5.4%). Finally, Hemminiki et al. [11] reported a higher risk of COPD in patients hospitalized with a number of different autoimmune disorders, including AS, compared to the general population, likely reflecting a general association between need for inpatient care and comorbidities.

In the present study, SpA patients with COPD were older and more likely to suffer from psoriasis, IBD and general comorbidities, compared to those without COPD. Taken together, this suggests that selection of patients with severe disease or worse general health in previous studies of AS explains the discrepant results and that the present study better reflects the prevalence of COPD among patients with SpA in general.

In contrast to COPD, we found higher proportions with a history of venous thromboembolism and cardiac arrythmia among patients with SpA compared to controls. Although investigation of these comorbidities was not the objective of this study, they have previously been associated with SpA, possibly reflecting shared disease mechanisms or exposures [8, 22]. For cardiac arrythmia, associations with long disease duration and active disease have been reported [24].

Limitations of the present study include the lack of validation of the SpA diagnoses, or the COPD outcome. However, previous studies of patients diagnosed with SpA overall [25] or with axSpA [26] at rheumatology units in the same region have demonstrated good validity for these diagnoses, similar to results for AS and undifferentiated SpA on a national level in Sweden [27]. Furthermore, the data on pharmacologic treatment, with 24% treated with TNF inhibitors currently or in the past, are largely compatible with the expected for a community-based cohort of patients with SpA, although a higher proportion (46%) has been reported in patients with axSpA followed at a rheumatology unit in the area [26]. The prevalence for SpA of 0.33% in the present study can be compared to a previous estimate from the same area for AS and undifferentiated SpA combined in 2007 among those aged ≥ 15 of 0.22% [25] and a national Swedish prevalence estimate for AS alone (age 16–64) from 2009 of 0.18% [28]. This indicates that the vast majority of patients with SpA overall should be included in the present study. On the other hand, the finding that 13% of those classified as SpA had received a diagnosis of RA during the study period suggests that some misclassification may have occurred.

Regarding COPD, we used several different definitions. Although the prevalence of COPD was lower when the definition included ≥ 1 diagnosis by a specialist in lung medicine, the ratio for those with SpA compared to controls was similar to the main analysis.

In addition, data on smoking and other life style factors were not available. Whereas smoking is a well-known risk factor for COPD, the relation between smoking and SpA is complex. There are conflicting data on the impact of smoking on the risk of developing SpA and on radiographic progression in patients with axSpA [29]. Confounding by other exposures is an issue in this context, and the present literature may be affected by citation bias [29]. A recent study of patients with early axSpA indicated that smokers may have higher disease activity over time [30]. In particular, there was an association for smoking with worse spinal pain and morning stiffness over time, adjusted for age, sex, level of education, other life style factors and time-varying treatment for axSpA [30]. Taken together, although more studies on this topic are needed, smoking may contribute both to SpA severity and to comorbidity with COPD.

On the other hand, the benefits of a healthy life style, including smoking cessation and regular physical activity, in patients with SpA are widely recognized and likely reflected in advice given by physicians, healthcare professionals and patient support groups. We cannot exclude that life style changes following SpA diagnosis may have contributed to a reduced prevalence of COPD in the present study.

Finally, although these data on prevalence provide important information on the relation between SpA and COPD, they do not give the whole picture. Further studies should investigate the incidence of COPD after diagnosis of SpA, taking important methodologic issues for sequential observational studies into account [31].

The assessment of COPD using data on primary and specialized care visits and dispensed prescriptions in an identical manner for SpA cases and controls is a strength of this study. As we used a comprehensive regional register, including data from primary care, the results should be generalizable. However, morbidity patterns in other populations may differ due to variability in ethnicity and environmental exposures.

In conclusion, the prevalence of COPD was not higher in patients with SpA compared to population controls in this community-based study, suggesting that there may not be an association between SpA and COPD.

## Supplementary Information

Below is the link to the electronic supplementary material.

Supplementary file1 (DOCX 23 kb)

## Data Availability

A limited dataset containing the data that support the main analyses is available from the corresponding author on reasonable request.
