# Supplementary material for: The prevalence of chronic obstructive pulmonary disease in patients with spondyloarthritis compared to the general population in the southernmost region of Sweden: a case–control study
Source: Clin Exp Med. 2024 Apr 10;24(1):75. doi: 10.1007/s10238-024-01335-x (PMC11006728; doi:10.1007/s10238-024-01335-x)
Supplement: Supplementary file 1 — Supplementary file1 (DOCX 23 kb) [file 10238_2024_1335_MOESM1_ESM.docx]

**Supplementary Tables**

**Supplementary Table 1.**

Overview of the ICD-10 and ATC codes used for the identification of COPD diagnosis and COPD medication, and ICD codes used for to identify the exposure (SpA)

|  | ICD-10 | ATC |
| --- | --- | --- |
| COPD | J41, J42, J43, J440, J441, J448, J449 | R03AC, R03BB, R03AK, R03AL, R03DX |
| SpA | M459, M460, M461, M468, M469 |  |

**Supplementary Table 2**.

Overview of ICD-10 and ATC codes used for identification of comorbidities

|  | ICD-10 | ATC |
| --- | --- | --- |
| Anterior uveitis | H20, H221 |  |
| Psoriasis | L40 |  |
| Inflammatory bowel disease | K50, K51 |  |
| Cardiovascular disease | I05, I06, I07, I08, I10, I11, I12, I13, I15, I20, I21, I22, I23, I24, I25, I27, I28, I34, I35, I36, I37, I50, I60, I61, I62, I63, I64, I65, I66, I67.0, I67.1, I67.2, I67.4, I68, I69, I70, I71, I72, I73.1, I73.9B, I73.9X, I74, I79, K55.0, K55.1, K55.9 | B01, C01, C02, C03, C07, C08, C09, C10 |
| Diabetes mellitus | E10, E11, E12, E13, E14, O24 | A10 |
| Venous thromboembolism | I26, I80, I81, I82 |  |
| Arrhythmia | I47, I48, I49 |  |

**Supplementary Table 3**

Chronic Obstructive Pulmonary Disease in patients with Spondyloarthritis and controls – base case definition, by sex and age.

|  | SpA cases with COPD  n / N | Controls with COPD  n / N | SpA | Controls | COPD Prevalence Ratio (95% CI) |
| --- | --- | --- | --- | --- | --- |
|  |  |  | COPD prevalence/1000 (95% CI) | COPD prevalence/1000 (95% CI) |  |
| Men – by age group | | | | | |
| <30 | 0 / 120 | 0 (0) | n/a | n/a | n/a |
| 30-39 | 0 / 256 | 5 / 1230 | n/a | 4.1 (1.3 – 9.5) | n/a |
| 40-49 | 2 / 372 | 5 / 1890 | 5.4 (0.7 – 19.4) | 2.6 (0.9 – 6.2) | 2.03  (0.19 – 21.20) |
| 50-59 | 6 / 410 | 38 / 2050 | 14.6 (5.4 – 31.9) | 18.5 (13.1 – 25.4) | 0.79  (0.27 – 1.88) |
| 60-69 | 15 / 441 | 92 / 1780 | 41.8 (23.4 – 68.9) | 51.7 (41.7 – 63.4) | 0.81  (0.43 – 1.40) |
| 70-79 | 18 / 234 | 103 / 1205 | 76.9 (45.6 – 121.6) | 85.5 (69.8 – 103.7) | 0.90  (0.51 – 1.49) |
| ≥80 | 14 / 82 | 61 / 465 | 170.7 (93.3 – 286.5) | 131.2 (100.3 – 168.5) | 1.30  (0.67 – 2.34) |
| Women – by age group | | | | | |
| <30 | 0 / 99 | 0 / 445 | n/a | n/a | n/a |
| 30-39 | 2 / 223 | 3 / 1085 | 9.0 (1.1 – 32.4) | 2.8 (0.6 – 8.1) | n/a |
| 40-49 | 0 / 403 | 21 / 2015 | n/a | 10.4 (6.5 – 15.9) | n/a |
| 50-59 | 15 / 441 | 61 / 2180 | 34.0 (19.0 – 56.1) | 28.0 (21.4 – 35.9) | 1.22  (0.64 – 2.15) |
| 60-69 | 26 / 319 | 115 / 1605 | 81.5 (53.2 – 119.4) | 71.7 (59.2 – 86.0) | 1.14  (0.71 – 1.75) |
| 70-79 | 28 / 188 | 121 / 1010 | 148.9 (99.0 – 215.3) | 119.8 (9.4 – 143.1) | 1.24  (0.79 – 1.88) |
| ≥80 | 9 / 65 | 32 / 350 | 138.5 (63.3 – 262.8) | 91.4 (62.5 – 129.1) | 1.51  (0.64 – 3.24) |

SpA: Spondyloarthritis, COPD: Chronic Obstructive Pulmonary disease, CI: Confidence interval; n/a not applicable

**Supplementary Table 4**

Chronic Obstructive Pulmonary Disease in patients with Spondyloarthritis and controls – strict case definition, by sex and age.

|  | SpA cases with COPD  n / N | Controls with COPD  n / N | SpA | Controls | COPD Prevalence Ratio (95% CI) |
| --- | --- | --- | --- | --- | --- |
|  |  |  | COPD prevalence/1000 (95% CI) | COPD prevalence/1000 (95% CI) |  |
| Men – by age group | | | | | |
| <30 | 0 / 120 | 0 / 545 | n/a | n/a | n/a |
| 30-39 | 0 / 256 | 4 / 1230 | n/a | 3.3 (0.9 – 8.3) | n/a |
| 40-49 | 0 / 372 | 1 / 1890 | n/a | 0.5 (0.0 – 2.9) | n/a |
| 50-59 | 5 / 410 | 19 / 2050 | 12.2 (4.0 – 28.5) | 9.3 (5.6 – 14.5) | 1.32  (0.38 – 3.70) |
| 60-69 | 9 / 359 | 69 / 1780 | 25.1 (11.5 – 47.6) | 38.8 (30.2 – 49.1) | 0.65  (0.28 – 1.30) |
| 70-79 | 14 / 234 | 70 / 1205 | 59.8 (32.7 – 100.4) | 58.1 (45.3 – 73.4) | 1.03  (0.54 – 1.84) |
| ≥80 | 10 / 82 | 45 / 465 | 122.0 (58.5 – 224.3) | 96.8 (70.6 – 129.5) | 1.26  (0.57 – 2.53) |
| Women – by age group | | | | | |
| <30 | 0 / 99 | 0 / 445 | n/a | n/a | n/a |
| 30-39 | 1 / 223 | 1 / 1085 | 4.5 (0.1 – 25.0) | 0.9 (0.0 – 5.1) | n/a |
| 40-49 | 0 / 403 | 15 / 2015 | n/a | 7.4 (4.2 – 12.3) | n/a |
| 50-59 | 11 / 441 | 41 / 2180 | 24.9 (12.5 – 44.6) | 18.8 (13.5 – 25.5) | 1.33  (0.61 – 2.62) |
| 60-69 | 18 / 319 | 78 / 1605 | 56.4 (33.4 – 89.2) | 46.8 (38.4 – 60.7) | 1.16  (0.65 – 1.95) |
| 70-79 | 19 / 188 | 88 / 1010 | 101.1 (60.8 – 157.8) | 87.1 (69.9 – 107.3) | 1.16  (0.67 – 1.91) |
| ≥80 | 7 / 65 | 24 / 350 | 107.7 (43.3 – 221.9) | 68.6 (43.9 – 102.0) | 1.57  (0.57 – 3.77) |

SpA: Spondyloarthritis, COPD: Chronic Obstructive Pulmonary disease, CI: Confidence interval; n/a not applicable

**Supplementary Table 5**

Chronic Obstructive Pulmonary Disease in patients with Spondyloarthritis and controls – strict case definition, with required diagnosis by specialist in lung medicine.

|  | SpA cases with COPD  n / N | Controls with COPD  n / N | SpA | Controls | COPD Prevalence Ratio (95% CI) |
| --- | --- | --- | --- | --- | --- |
|  |  |  | COPD prevalence/1000 (95% CI) | COPD prevalence/1000 (95% CI) |  |
| All | 39/3571 | 177/17855 | 10.9 (7.8-14.9) | 9.9 (8.5-11.5) | 1.10  (0.76-1.56) |
| Women | 18/1738 | 90/8690 | 10.4 (6.1-16.4) | 10.4 (8.3-12.7) | 1.00  (0.57-1.66) |
| Men | 21/1833 | 87/9165 | 11.5 (7.1-17.5) | 9.5 (7.6-11.7) | 1.21  (0.71-1.95) |
| All – by age group (years) | | | | | |
| <30 | 0 / 219 | 0/990 | n/a | n/a | n/a |
| 30-39 | 0 / 479 | 0/2315 | n/a | n/a | n/a |
| 40-49 | 0 / 775 | 2/3905 | n/a | 0.5 (0.1-0.9) | n/a |
| 50-59 | 7/851 | 21/4230 | 8.2 (3.3-16.9) | 5.0 (3.1-7.6) | 1.66  (0.60-4.07) |
| 60-69 | 8/678 | 50/3385 | 11.8 (5.1-23.2) | 14.8 (11.0-19.5) | 0.80  (0.33-1.69) |
| 70-79 | 14/422 | 72/2215 | 33.2 (18.1-55.7) | 32.5 (25.4-40.9) | 1.02  (0.53-1.82) |
| ≥80 | 10/147 | 32/815 | 68.0 (32.6-125.1) | 39.3 (26.9-55.4) | 1.73  (0.76-3.60) |

SpA: Spondyloarthritis, COPD: Chronic Obstructive Pulmonary disease, CI: Confidence interval; n/a not applicable
